# Predictors for blood loss and transfusion frequency to guide blood saving programs in primary knee- and hip-arthroplasty

**DOI:** 10.1038/s41598-021-82779-z

**Published:** 2021-02-23

**Authors:** Christina Pempe, Robert Werdehausen, Philip Pieroh, Martin Federbusch, Sirak Petros, Reinhard Henschler, Andreas Roth, Christian Pfrepper

**Affiliations:** 1 Department of Orthopedics, Trauma and Plastic Surgery, University Hospital Leipzig, Leipzig, Germany; 2 Department of Anesthesiology and Intensive Care, University Hospital Leipzig, Leipzig, Germany; 3 Institute of Laboratory Medicine, University Hospital Leipzig, Leipzig, Germany; 4 Division of Hemostaseology, Department of Hematology, Cellular Therapy and Hemostaseology, University Hospital Leipzig, Liebigstr. 20, 04103 Leipzig, Germany; 5 Medical ICU, University Hospital Leipzig, Leipzig, Germany; 6 Institute of Transfusion Medicine, University Hospital Leipzig, Leipzig, Germany

**Keywords:** Medical research, Risk factors

## Abstract

Endoprosthetic surgery can lead to relevant blood loss resulting in red blood cell (RBC) transfusions. This study aimed to identify risk factors for blood loss and RBC transfusion that enable the prediction of an individualized transfusion probability to guide preoperative RBC provision and blood saving programs. A retrospective analysis of patients who underwent primary hip or knee arthroplasty was performed. Risk factors for blood loss and transfusions were identified and transfusion probabilities computed. The number needed to treat (NNT) of a potential correction of preoperative anemia with iron substitution for the prevention of RBC transfusion was calculated. A total of 308 patients were included, of whom 12 (3.9%) received RBC transfusions. Factors influencing the maximum hemoglobin drop were the use of drain, tranexamic acid, duration of surgery, anticoagulation, BMI, ASA status and mechanical heart valves. In multivariate analysis, the use of a drain, low preoperative Hb and mechanical heart valves were predictors for RBC transfusions. The transfusion probability of patients with a hemoglobin of 9.0–10.0 g/dL, 10.0–11.0 g/dL, 11.0–12.0 g/dL and 12.0–13.0 g/dL was 100%, 33.3%, 10% and 5.6%, and the NNT 1.5, 4.3, 22.7 and 17.3, while it was 100%, 50%, 25% and 14.3% with a NNT of 2.0, 4.0, 9.3 and 7.0 in patients with a drain, respectively. Preoperative anemia and the insertion of drains are more predictive for RBC transfusions than the use of tranexamic acid. Based on this, a personalized transfusion probability can be computed, that may help to identify patients who could benefit from blood saving programs.

## Introduction

Total knee arthroplasty (TKA) and total hip arthroplasty (THA) belong to the most frequently performed orthopedic procedures worldwide. They can result in relevant blood loss and up to 46% of patients require red blood cell (RBC) transfusions during or following surgery^1–6^. Despite major improvements in blood transfusion safety in the last years, allogenic blood transfusion can still lead to several complications, including allergic reactions, infections^7^, circulatory overload and transfusion-associated lung injury^2^. In addition, blood transfusions can result in prolonged hospitalization, higher mortality and morbidity and may increase the risk for surgical site infections^8,9^. Preoperative anemia is a common condition in patients undergoing major surgery. A meta-analysis has shown an overall prevalence rate of preoperative anemia in major surgery of 39.1%^10^. Preoperative anemia was prevalent in approximately 25% of the patients admitted for elective orthopedic surgery^8,11^. Severe anemia is associated with increased mortality and higher incidence for RBC transfusions^10^. Iron deficiency anemia is the most common type of anemia in patients undergoing arthroplasty and it is associated with longer hospital stay, higher 90 days readmission rates and higher incidence of postoperative complications^12,13^.

Tranexamic acid (TXA) is a synthetic derivative of the amino acid lysine, which inhibits lysine binding to plasminogen and thus blocks its conversion to plasmin^14^. Recent literature demonstrated the effectiveness of intravenous tranexamic acid to reduce blood loss and transfusions rates in patients undergoing total hip and knee arthroplasty^5,15–17^. Patients receiving tranexamic acid had no increased risk for complications, such as thromboembolic events or acute kidney failure^18,19^.

In the present study we analyzed factors predicting blood loss and transfusion requirement after elective hip and knee arthroplasty with a special focus on the use of tranexamic acid (TXA), requirement of anticoagulation and preoperative conditions like anemia and impaired renal function.

## Methods

### Patient selection

All patients who underwent primary total hip arthroplasty or total knee arthroplasty at the University Hospital Leipzig between June 2018 and November 2019 were retrospectively analyzed. Starting from January 2019, all patients without contraindications received prophylactic infusion of TXA (1000 mg) 30 min prior to the beginning of the surgery as per institutional protocol. Contraindications for the administration of TXA were history of venous thromboembolism, severe renal dysfunction (GFR < 30 mL/min), known epilepsy, coronary artery disease, history of stroke or intake of anticoagulants. Surgery was performed by senior consultants specialized in arthroplasty using the same prosthesis designs. All patients without an indication for therapeutic anticoagulation received prophylactic low molecular weight heparin subcutaneously starting from 6 h after surgery, for 30 days (hip arthroplasty) or 14 days (knee arthroplasty) after surgery.

The following variables were collected for every patient: age, gender, body mass index, duration of surgery, American Society of Anesthesiologists (ASA) grade, Metabolic Equivalent of Task (MET) score and intraoperative insertion of suction drains. Hemoglobin levels were measured preoperatively and one and three to five days postoperatively. Blood transfusions were administered in patients with a Hb ≤ 6 g/dL or in patients with a Hb ≤ 8 g/dL presenting with symptoms of inadequate oxygen delivery, such as congestive heart failure, hypotension, tachycardia, chest pain or dyspnea. Based on our internal policy and recommendations for autologous hemotherapy^20^ prophylactic allocation of RBC was performed for procedures with a transfusion probability of ≥ 10% but no prophylactic collection of autologous RBCs was performed in any patient.

The mean blood loss was calculated with the Meunier’s^21^ and Lopez-Picard equations^22^. The blood volume was estimated according to the Nadler’s formula^23^ for the Meunier’s equation and with to the formula of the International Council for Standardization in Hematology (ICSH)^24^ for the Lopez-Picard equation.

### Statistics

Data are given as median with interquartile range (IQR) in brackets. The distributions of all variables were tested with Kolmogorov–Smirnov-test. A t-test was used to compare means if normal distribution of variables could be assumed, otherwise the Mann–Whitney U test was performed. Multivariate analysis for the prediction of blood transfusion was made using a binary logistic regression model. The odds ratio (OR) and the 95% confidence interval was calculated. The level of significance was set at p < 0.05. Statistical analyses were performed using the software SPSS version 26 (SPSS Inc, Chicago, IL).

### Ethical considerations

The study was approved by the Ethical committee of the University of Leipzig (reference 178/20-ek), and conducted according to the Declaration of Helsinki. The need for informed consent was waived by the Ethical Committee at the Medical Faculty of the Leipzig University, Germany, but according to the Ethical approval, patients had to be excluded who refused to have their data used for research purposes under the admission contract with the Leipzig University Hospital. During the observation period, no patient refused to have their data used for research purposes.

## Results

A total of 308 patients were included into the analysis. Median age was 66 (IQR 58–76) years, and 53.6% were female. Total hip arthroplasty was carried out in 202 patients (69.5%), while 79 patients (25.6%) underwent a total knee arthroplasty. A minimal invasive anterolateral approach was used in 75 (37.1%) patients receiving a total hip arthroplasty. A fully cemented knee arthroplasty was performed in 58 (73.4%), a hybrid operation (uncemented femoral and cemented tibial component) in 18 (22.8%) and a cementless arthroplasty in 3 (3.8%) patients. Twenty-five patients received unicondylar knee arthroplasty and two patients underwent an isolated replacement of the femoropatellar joint. A total of 87 (28.2%) patients were under anticoagulation, predominantly platelet aggregation inhibitors (n = 49, 15.9%). The median duration of surgery was 73 (61–90) minutes, without a significant difference between knee and hip arthroplasty.

Patient characteristics are given in Table 1.

**Table 1:** Patient characteristics.

Female gender	%	53.6
Age (years)	Median (IQR)	66 (58–76)
BMI (kg/m^2^)	Median (IQR)	27.9 (24.9–32.0)
**ASA**		
I	n (%)	24 (7.8)
II	n (%)	214 (69.5)
III	n (%)	66 (21.4)
IV	n (%)	3 (1.0)
**MET**		
< 5	n (%)	126 (46.7)
≥ 5	n (%)	144 (53.3)
**Surgery**		
Total hip arthroplasty	n (%)	202 (65.6)
Total knee arthroplasty	n (%)	79 (25.6)
unicondylar arthroplasty	n (%)	25 (8.1)
Wave	n (%)	2 (0.6)
**Anticoagulation**		
No anticoagulation	n (%)	221 (71.8)
Platelet inhibitor	n (%)	49 (15.9)
DOAC	n (%)	27 (8.8)
Phenprocoumon	n (%)	11 (3.6)
Drain inserted	n (%)	136 (44.2)
Use of tranexamic acid	n (%)	118 (38.3)

*BMI* body mass index, *ASA* American Society of Anesthesiologists grade, *MET* Metabolic Equivalent of Task score.

Patients undergoing knee arthroplasty had a higher median body weight (90 vs. 78 kg, p < 0.001) and a higher BMI (31.7 vs. 27.8 kg/m^2^, p < 0.001) than patients with hip arthroplasty, resulting in a higher blood volume according to Nadler’s formula: 5091 vs. 4695 ml (p = 0.001) and the ICSH formula: 4919 vs. 4653 mL (p = 0.02).

### Perioperative hemoglobin values

Overall, the median preoperative Hb was 13.9 (12.7–14.7) g/dL. Significantly lower Hb was found in women than in men (13.0 g/dL vs. 14.3 g/dL, p < 0.001), as well as in patients > 66 years old than in patients ≤ 66 years (13.2 g/dL vs.14.0 g/dL, p < 0.001). Patients with an ASA of > 2 also had lower Hb than those with an ASA ≤ 2 (13.0 vs. ASA: 13.9 g/dL, p = 0.008). The Hb level was also lower in patients with a MET score of < 5 compared to those with a score ≥ 5 (13.1 vs. 14.0, p = 0.001). There was a trend towards a lower Hb in patients receiving therapeutic anticoagulation than in those without this therapy (13.8 g/dL vs.13.5 g/dL, p = 0.063). The preoperative Hb did not correlate with BMI (BMI < 28 kg/m^2^: 13.7 g/dL vs. BMI > 28 kg/m^2^ 13.7 g/dL). Patients with a preoperative Hb < 12 g/dL had a lower GFR (64.0 [47.5–91.0] vs. 84.0 [69–94] mL/min, p = 0.004), a lower mean corpuscular hemoglobin (MCH: 1.83 [1.77–1.88] vs. 1.88 [1.81–1.94] fmol, p = 0.009) and a lower mean corpuscular hemoglobin concentration (MCHC: 21.2 [20.7–21.4] vs. 21.5 [21.1–22.0] mmol/L, p = 0.001) than those with a Hb of > 12 g/dl. In addition, the proportion of patients with a GFR, MCH and MCHC below the reference range was higher in patients with low Hb (Supplementary Table 1). There were no patients with a mean corpuscular volume (MCV) below the reference range in our cohort.

The median drop in Hb between the pre-operative value and the first postoperative day was 2.6 (IQR 1.8–3.7) g/dL and the maximum drop in Hb postoperatively was 3.9 (IQR 2.6–4.8) g/dL. The maximum drop in Hb was not influenced by the type of operation (knee vs. hip arthroplasty: 4.2 (IQR 3.2–5.3) vs. 4.0 (IQR 3.1–4.8) g/dL, p = 0.223), age and MET score. Patients receiving anticoagulants had a higher maximum drop in Hb but there was no difference between different types of anticoagulants. Factors influencing the maximum drop in Hb are shown in Table 2.

**Table 2:** Factors influencing the maximum drop in hemoglobin.

	Maximum hemoglobin drop (g/dL)		p
	Median	IQR	
**Drain**			
Yes	4.35	3.54–5.32	< 0.001
No	3.38	2.26–4.35	
**Tranexamic acid**			
Yes	3.38	2.42–4.19	< 0.001
No	4.19	2.90–5.00	
**Tranexamic acid**			
Drain	4.11	3.18–4.71	< 0.001
No drain	3.14	2.09–3.87	
**No tranexamic acid**			
Drain	4.51	3.54–5.48	
No drain	3.54	2.42–4.67	
**Anticoagulation**			
Yes	4.35	3.06–5.00	0.004
No	3.54	2.50–4.59	
**Duration of surgery**			
≤ 73 min (median)	3.55	2.42–4.51	0.002
> 73 min (median)	4.08	3.06–5.00	
**BMI**			
< 28 (median)	4.03	3.06–5.00	0.024
≥ 28 (median)	3.54	2.42–4.67	
**ASA status**			
≤ 2	3.71	2.58–4.67	0.016
> 2	4.19	3.14–5.24	
**Mechanical heart valve**			
Yes	5.96	4.35–n.a.	0.034
No	3.87	2.58–4.83	

BMI: body mass index, ASA: American Society of Anesthesiologists graden, n.a.: not applicable.

### Calculation of perioperative blood loss

The mean blood loss calculated with the Meunier’s formula was higher compared to the Lopez-Picard formula (1949 mL vs. 1496 mL), but there was a high correlation between the results for each patient (Spearman r = 0.902, p < 0.001). The perioperative blood loss was lower in patients receiving hip compared to knee arthroplasty, in patients without a suction drain, in patients receiving tranexamic acid, in patients with a lower Hb, in non-anticoagulated patients and in patients with low ASA status.

### The role of anticoagulation

Patients on anticoagulation (either platelet inhibitor, direct oral anticoagulant or vitamin K antagonist) were older (median 75 vs. 64 years, p < 0.001), had a higher maximum drop in Hb (median 4.4 vs. 3.5 g/dL, p = 0.004) and were longer hospitalized postoperatively (median 7 vs. 6 days, p = 0.014) than those without any anticoagulant treatment. Patients receiving VKA had a higher transfusion rate compared to patients on DOAC (p = 0.032) and to patients with platelet inhibitors (p = 0.012). There were three patients with mechanical heart valve replacement (aortic valve, n = 2 and mitral valve, n = 1) receiving VKA. Two of these patients required RBC transfusions. The preoperative Hb was 10.9 and 14.8 g/dL in the two patients receiving RBC transfusions and 15.5 g/dL in the patient without RBC transfusion.

### Transfusion requirement

A total of 12 (3.9%) patients received RBC transfusions. In one patient, a single RBC unit was transfused, eight patients received two units, two patients received four RBCs and six RBCs were administered in one patient. The median postoperative hospitalization was 7 days in patients receiving transfusions vs. 6 days in patients without transfusions, without reaching statistical significance (p = 0.12). Factors influencing RBC transfusions are displayed in Table 3.

**Table 3:** Factors influencing red blood cell (RBC) transfusions. Values are given as median (interquartile range).

	RBC transfusion, n = 12	No RBC transfusion, n = 296	p
Age (years)	83 (64–85)	66 (58–76)	0.009
BMI (kg/m^2^)	24.2 (22.5–28.0)	28.0 (25.0–32.2)	0.006
Preoperative hemoglobin (g/dL)	11.7 (11.0–12.6)	13.9 (12.7–14.8)	< 0.001
Hemoglobin level on POD 1 (g/dL)	7.5 (7.0–9.4)	11.0 (10.2–11.9)	< 0.001
Hemoglobin level on POD 3–5 (g/dL)	6.6 (6.3–8.3)	9.7 (8.2–11.0)	0.001
Maximum drop in hemoglobin (g/dL)	4.8 (4.2–5.8)	3.9 (2.6–4.8)	0.036
Drop in hemoglobin between preoperative value and POD 1 (g/dL)	3.4 (2.7–5.0)	2.7 (1.8–3.7)	0.037
Hospitalization after operation (days)	7.0 (6.3–7.8)	6.0 (5.0–7.0)	0.12

*POD* postoperative day.

Age ≥ 75 years, BMI < 30 kg/m^2^, ASA status > 2, the use of drains, the avoidance of tranexamic acid, a low preoperative Hb and an MCHC below the lower limit of normal (LLN) were associated with a significantly higher transfusion frequency. The transfusion frequency in different cohorts of patients and predictors for transfusions are shown in Table 4.

**Table 4:** Transfusion frequency in different patient cohorts.

Predictor	n	%	p
**Age**			
≤ 66 years (median)	3/157	1.9	0.066
> 66 years (median)	9/151	6.0	
< 75 years	5/217	2.3	0.026
≥ 75 years	7/91	7.7	
**BMI**			
< 28 (median)	9/155	5.8	0.081
≥ 28 (median)	3/153	2.0	
< 30	12/196	6.1	0.008
≥ 30	0/112	0.0	
**ASA**			
≤ 2	6/238	2.5	0.02
> 2	6/69	8.7	
**Anticoagulation**			
Yes	6/87	6.9	0.088
No	6/221	2.7	
**Anticoagulation**			
No	6/221	2.7	0.001
Platelet inhibitor	2/49	4.1	
DOAC	1/27	3.7	
VKA	3/11	27.3	
**Mechanical heart valve**			
Yes	2/3	66.7	0.004
No	10/305	3.3	
**Operation**			
Knee arthroplasty	2/79	2.5	0.367
Hip arthroplasty	10/202	5.0	
**Hip arthroplasty**			
Standard lateral approach	8/127	6.3	0.328
Minimal invasive anterolateral approach	2/75	2.7	
**Drains**			
Yes	11/136	8.1	0.001
No	1/171	0.6	
**Tranexamic acid**			
Yes	1/118	0.8	0.029
No	11/190	5.8	
**Tranexamic acid**			
Drain	1/38	2.6	0.001
No drain	0/80	0.0	
**No tranexamic acid**			
Drain	10/98	10.2	
No drain	1/91	1.1	
**Hemoglobin < 11.0 g/dL**			
Yes	4/6	66.7	< 0.001
No	8/302	2.6	
**Hemoglobin < 12.0 g/dl**			
Yes	6/26	23.1	< 0.001
No	6/282	2.1	
**Hemoglobin < 13.0 g/dL**			
Yes	10/97	10.3	< 0.001
No	2/211	0.9	
**Hemoglobin < 14.0 g/dL**			
Yes	10/165	6.1	0.035
No	2/143	1.4	
**GFR < 60 mL/min**			
Yes	3/47	6.4	0.348
No	9/258	3.5	
**MCHC < LLN**			
Yes	2/5	40.0	0.013
No	10/303	3.3	

*LLN* lower limit of normal.

### Multivariate analysis

A multivariate analysis including the univariate risk factors: use of drain, use of tranexamic acid, contraindications for the use of tranexamic acid, lower preoperative hemoglobin and preoperative hemoglobin < 13 g/dL, older age and age ≥ 75 years, lower BMI and BMI < 30 kg/m^2^, ASA status > 2, duration of the operation > 73 min, the use of anticoagulants, the use of VKA and existence of mechanical heart valve was performed. In multivariate analysis, only the use of drain (OR: 0.053 [95% 0.006–0.489], p = 0.01), lower preoperative hemoglobin level (OR: 0.205 [95% CI 0.090–0.465], p < 0.001) and the existence of a mechanical heart valve (OR: 0.002 [95% CI 0.000–0.080], p = 0.001) were significant risk factors for transfusions. After exclusion of patients with mechanical heart valves, only the use of drain (OR: 0.054 [95% 0.006–0.496], p = 0.01) and lower preoperative hemoglobin level (OR: 0.214 [95% CI 0.096–0.477], p < 0.001) persisted as multivariate risk factors.

### Patient blood management

The transfusion frequency in different patient cohorts with different preoperative Hb ranges was calculated. The threshold for prophylactic allocation of RBC of ≥ 10% is reached at a hemoglobin level of 13.2 g/dL in the total cohort, at 14.0 g/dL in patients not receiving tranexamic acid and at 14.8 g/dL in patients in whom a suction drain is used. Based on this threshold and preoperative hemoglobin values, prophylactic allocation of RBC would not be necessary in 189 patients (61.4%). Taking into account a planned use of tranexamic acid and the preoperative hemoglobin levels, 10 of 118 (8.5%) patients receiving tranexamic acid, but 108 of 190 (56.8%) patients not receiving tranexamic acid have a transfusion probability of ≥ 10%. As the transfusion frequency in patients not receiving a drain was only 0.6%, prophylactic provision is not necessary in these patients, while 101 of 136 (74.3%) patients with a drain had a transfusion frequency > 10%.

The number needed to treat (NNT) for the reduction of transfusion requirement was determined based on the fact that with the infusion of 1000 mg iron isomaltoside, a median increase in hemoglobin level of 1.0 g/dL within four weeks can be achieved^25^. The transfusion frequency among patients with a preoperative Hb < 11 g/dL was 67%. The theoretical NNTs for the whole cohort of patients with a hemoglobin level of 9.0–10.0 g/dL, 10.0–11.0 g/dL and 11.0–12.0 g/dL was 1.5, 4.3 and 22.7, respectively. The transfusion frequency and NNTs for all patients and different subgroups of high-risk patients are given in Table 5. Patients with mechanical heart valves were excluded from the calculation due to their high transfusion frequency irrespective of the preoperative Hb.

**Table 5:** Transfusion frequency in patients with different pre-operative hemoglobin levels and predicted number needed to treat (NNT) to prevent red blood cell transfusion by an iron infusion-induced increase of the preoperative hemoglobin of 1 g/dL.

Hemoglobin (g/dL)	All Patients, n = 305			Patients not receiving tranexamic acid, n = 187			Patients receiving a drainage, n = 133			Patients not receiving tranexamic acid, but a drainage, n = 95		
	n	%	NNT	n	%	NNT	n	%	NNT	n	%	NNT
9.0–10.0	2/2	100.0	1.5	1/1	100.0	2.0	2/2	100.0	2.0	1/1	100.0	2.0
10.0–11.0	1/3	33.3	4.3	1/2	50.0	3.0	1/2	50.0	4.0	1/2	50.0	6.0
11.0–12.0	2/20	10.0	22.7	2/12	16.7	11.9	1/4	25.0	9.3	1/3	33.3	6.0
12.0–13.0	4/71	5.6	17.9	4/48	8.3	12.0	4/28	14.3	7.0	4/24	16.7	6.0
13.0–14.0	0/68	0.0	n.a.	0/35	0.0	n.a.	0/32	0.0	n.a.	0/19	0.0	n.a.
14.0–15.0	1/86	1.2	86.0	1/54	1.9	54.0	1/40	2.5	40.0	1/29	3.4	29.0
> 15.0	0/55	0.0	n.a.	0/35	0.0	n.a.	0/25	0.0	n.a.	0/17	0.0	n.a.

*n.a.* not applicable.

## Discussion

The aim of this study was to investigate factors affecting perioperative hemoglobin loss and transfusion frequency in patients following hip and knee arthroplasty. We identified the use of drain a lower preoperative Hb and the existence of a mechanical heart valve as independent predictors for RBC transfusions. According to our institutional protocol and recent guidelines^26^, patients with mechanical heart valves receive a perioperative anticoagulation with continuous infusion of unfractionated heparin at a therapeutic dose starting early after the operation when hemostasis is achieved. It is well known that heparin bridging of anticoagulation with VKA leads to an increased risk of perioperative bleeding events^27,28^ and in patients after implantation of mechanical heart valves^29,30^. Therefore, the higher transfusion frequency in patients with mechanical heart valves and in patients receiving anticoagulation is well explained by the bridging therapy.

### Predictors for blood loss and transfusion

The insertion of drains after knee or hip arthroplasty is still a matter of controversy^31–33^. The insertion of a suction drain has no scientifically proven benefits and drains are not used routinely anymore. Using enhanced recovery protocols in the arthroplasty leads to a changing role for drains, particularly with the use of tranexamic acid. Most literature is from the pre-tranexamic era and there are only few studies on the use of drains in combination with tranexamic acid. In our cohort, the use of suction drains was associated with a greater Hb drop and was an independent risk factor for higher transfusion rates (8.1% vs. 0.6%). Similar results were published by Concina et al. who have also found a decrease in Hb and a lower transfusion frequency in total knee arthroplasty^34^. Although Chen et al. determined a higher Hb drop in patients after knee arthroplasty with a drain but no higher transfusion frequency^35^. Others found no correlation between the use of drains and Hb drop^31,36^, but the number of patients included in these last two studies was much lower than in the cohort reported by Chen and colleagues. In our study, none of the 80 patients in whom tranexamic acid was used and suction drains were avoided received a blood transfusion. In contrast, the transfusion rate was 10.2% in patients not receiving tranexamic acid but a suction drain.

Tranexamic acid has been proved to be effective in preventing blood transfusions as well as reducing perioperative blood loss. Recent meta-analyses reported on reduced blood loss and lower transfusion rates for patients receiving tranexamic acid, without an increased risk for deep vein thrombosis, pulmonary embolism or other complications^37,38^. These findings are consistent with our results. However, our data indicate that the avoidance of suction drains might be as important as the use of tranexamic acid for the reduction of perioperative blood loss.

Preoperative anemia is common in orthopedic surgery and is prevalent in approximately 25% of patients^8,11^. Low preoperative Hb has been identified as an independent risk factor for postoperative transfusions^39–41^. Ryan et al. reported a Hb of 12.5 g/dL as an optimal cutoff for predicting postoperative transfusion with a specificity 76.4% and a sensitivity of 84.8%^42^. We found a transfusion probability of > 10% at a hemoglobin level of 13.2 g/dL in the total cohort, at 14.0 g/dL in patients not receiving tranexamic acid and at 14.8 g/dL in patients in whom a suction drain was used. Based on our internal policy and recommendations for autologous hemotherapy^43^, prophylactic allocation of RBC is recommended for procedures with a transfusion probability of ≥ 10%. Taking into account a planned use of tranexamic acid and the pre-operative hemoglobin level, only 10 of 118 (8.5%) patients receiving tranexamic acid but 108 of 190 (56.8%) patients not receiving tranexamic acid have a transfusion probability of ≥ 10% and therefore require prophylactic provision of packed RBC according to our policy. As the transfusion frequency in patients not receiving a suction drain was only 0.6%, prophylactic provision of RBC units is not necessary in these patients; but 74.3% of patients with a drainage had a transfusion probability > 10% based on their pre-operative hemoglobin level. Based on these data a personalized transfusion probability can be calculated, resulting in a further reduction of prophylactic RBC provision.

### Patient blood management

Perioperative blood optimization programs have been developed in the last years to reduce transfusion frequency and to increase patient safety. These programs include the pre-operative optimization of the hemoglobin level and the optimal hemostasis obtained during surgery. The comparably low transfusion rate of 3.9% observed in our cohort might be in part explained with the use of minimal invasive surgery in total hip arthroplasty. Various studies have shown a significant lower blood loss by using the minimal invasive anterolateral compared to the standard lateral approach due to smaller skin and muscle incisions^44,45^. In our cohort, patients who received a minimal invasive approach had a transfusion rate of only 2.7%. In addition, most patients undergoing knee arthroplasty received a cemented prosthesis which causes lower blood loss compared to a cementless arthroplasty^46^. Optimal hemostasis during surgery was maintained by avoiding hypothermia and optimization of pH level. A meta-analysis reported that even mild hypothermia significantly increases blood loss and the risk for transfusion by 22%^47^. In addition, RBC transfusions were administered restrictively only in patients with Hb ≤ 6 g/dL or in symptomatic patients with Hb ≤ 8 g/dL.

Kopanidis et al. achieved a lower blood transfusion rate and higher postoperative hemoglobin values after implementing a patient blood management program^48^. Pinilla-Gracia et al. also showed lower transfusion rates, shorter length of stay and a corrected preoperative anemia in 79% of cases^49^. Prevention of postoperative anemia is a fundamental issue to avoid blood transfusions and potential complications and also to save costs. Fenelon reported on a 46% reduction of cross-matched blood and an annual cost saving of €54,375 after introduction of an enhanced recovery program^50^. Apart from cost saving, supplementation with iron isomaltoside is very well tolerated with a number needed to harm for side effects of 15–25 and for serious side effects > 500^51^ resulting in a positive risk–benefit ratio especially in patients with additional risk factors and a low NNT. In addition, the co-administration of erythropoietin has shown to further reduce blood transfusions in patients undergoing major orthopedic surgery^52^. Several studies have reported that blood transfusions are associated with an increased perioperative mortality, more complications, especially surgical site infections and prolonged hospitalization^3,10,53–55^. Therefore, the inclusion of these patients in preoperative blood saving programs and pre-operative adjustment of iron deficiency anemia is highly warranted. According to our data, particularly patients with a low preoperative hemoglobin level who are not receiving tranexamic acid and a suction drain is used, are at high risk and should be included in such programs.

In addition, we were able to show that a personalized estimation of the transfusion probability can be calculated. This formula will be implemented in the clinical decision support system AMPEL (Analysis and Monitoring System for Patient Safety by Enhanced Laboratory Decision Support), which is used at the University Hospital Leipzig^56^ to allow for an automatized identification of patients with increased risk for postoperative RBC transfusions.

### Limitations

The present study has several limitations, mainly due to its retrospective design. Secondly, patients have been operated by different surgeons, which might have influenced bleeding. Thirdly, the criteria for insertion of suction drains were not standardized. Thus, surgeons’ expertise and patient risk factors may have influenced the decision on the use of suction drains. Finally, tranexamic acid was not given in patients on therapeutic anticoagulation, resulting in a selection bias. However, as the use of anticoagulants, apart from patients with mechanical heart valves, was not predictive for transfusions in the multivariate analysis, this bias seems to be of limited relevance.

## Conclusion

Preoperative anemia and the insertion of drains are the strongest predictors for RBC transfusion requirement in our analysis and turned out to be even more predictive than the well-established use of tranexamic acid. Therefore, the insertion of drains should be avoided and patients should be screened for preoperative anemia. In addition, the calculation of a personalized transfusion probability is feasible and may enable automatized clinical decision systems with regard to preoperative packed red cells allocation and other blood patient management options such as iron substitution. This could contribute to save resources, reduce costs and enhance patient safety.

## Supplementary Information

Supplementary Information.
