# Supplementary material for: Predictors for blood loss and transfusion frequency to guide blood saving programs in primary knee- and hip-arthroplasty
Source: Sci Rep. 2021 Feb 23;11:4386. doi: 10.1038/s41598-021-82779-z (PMC7902666; doi:10.1038/s41598-021-82779-z)
Supplement: Supplementary file 1 — Supplementary Information. [file 41598_2021_82779_MOESM1_ESM.docx]

**Predictors for blood loss and transfusion frequency to guide blood saving programs in primary knee- and hip- arthroplasty**

Christina Pempe ^1^, Robert Werdehausen ^2^, Philipp Pieroh ^1^, Martin Federbusch ^3^, Sirak Petros ^4, 5^, Reinhard Henschler ^6^, Andreas Roth ^1^, Christian Pfrepper ^4^

^1^ Department of Orthopedics, Trauma and Plastic Surgery, University Hospital Leipzig, Leipzig, Germany

^2^ Department of Anesthesiology and Intensive Care, University Hospital Leipzig, Leipzig, Germany

^3^ Institute of Laboratory Medicine, University Hospital Leipzig, Leipzig, Germany

^4^ Division of Hemostaseology, Department of Hematology, Cellular Therapy and Hemostaseology, University Hospital Leipzig, Leipzig, Germany

^5^ Medical ICU, University Hospital Leipzig, Leipzig, Germany

^6^ Institute of Transfusion Medicine, University Hospital Leipzig, Leipzig, Germany.

Supplementary figure 1: Proportion of patients with laboratory values below the reference range and different hemoglobin levels.

| Value | Hemoglobin level | n | % | p |
| --- | --- | --- | --- | --- |
| GFR < 60 ml/min | < 11.0 g/dL | 3/6 | 50% | <0.049 |
|  | > 11.0 g/dL | 44/299 | 14.7% |  |
|  | < 12.0 g/dL | 11/25 | 44.0% | <0.001 |
|  | > 12.0 g/dL | 36/280 | 12.9 % |  |
|  | < 13.0 g/dL | 31/96 | 32.3 % | <0.001 |
|  | > 13.0 g/dL | 16/209 | 7.7 % |  |
|  | < 14.0 g/dL | 37/164 | 22.6 % | <0.001 |
|  | > 14.0 g/dL | 10/141 | 7.1% % |  |
| MCH < LLN | < 11.0 g/dL | 1/6 | 16.7% | 0.361 |
|  | > 11.0 g/dL | 21/302 | 7.0% |  |
|  | < 12.0 g/dL | 4/26 | 15.4% | 0.102 |
|  | > 12.0 g/dL | 18/282 | 6.4 % |  |
|  | < 13.0 g/dL | 14/97 | 14.4 % | 0.001 |
|  | > 13.0 g/dL | 8/211 | 3.8 % |  |
|  | < 14.0 g/dL | 19/165 | 11.5 % | 0.001 |
|  | > 14.0 g/dL | 3/143 | 2.1% % |  |
| MCHC < LLN | < 11.0 g/dL | 2/6 | 33.3% | 0.003 |
|  | > 11.0 g/dL | 3/302 | 1.0% |  |
|  | < 12.0 g/dL | 3/26 | 11.5% | 0.005 |
|  | > 12.0 g/dL | 2/282 | 0.7 % |  |
|  | < 13.0 g/dL | 5/97 | 5.2 % | 0.003 |
|  | > 13.0 g/dL | 0/211 | 0.0 % |  |
|  | < 14.0 g/dL | 5/165 | 3.0 % | 0.064 |
|  | > 14.0 g/dL | 0/143 | 0.0% |  |

GFR: Glomerular filtration rate, LLN: lower limit of normal
